# Cross-Cultural Adaptation and Psychometric Properties of the Swahili Version of the European Organization for Research and Treatment of Cancer (EORTC) QLQ-BR45 among Breast Cancer Patients in Tanzania

**DOI:** 10.3390/healthcare11182467

**Published:** 2023-09-05

**Authors:** Paulo L. Kidayi, Amir H. Pakpour, Fredrik Saboonchi, Freddie Bray, Hélio Manhica, Christina C. Mtuya, Furaha Serventi, Ragnhild E. Aune, Michael J. Mahande, Gunilla Björling

**Affiliations:** 1 Faculty of Nursing, Kilimanjaro Christian Medical University College, Moshi 2240, Tanzania; paulo.kidayi@kcmuco.ac.tz (P.L.K.); christina.mtuya@kcmuco.ac.tz (C.C.M.); michael.mahande@kcmuco.ac.tz (M.J.M.); 2 Department of Nursing, School of Health and Welfare, Jönköping University, SE-55111 Jönköping, Sweden; amir.pakpour@ju.se; 3 Department of Health Sciences, Swedish Red Cross University, SE-17176 Stockholm, Sweden; fredrik.saboonchi@ki.se; 4 Division of Insurance Medicine, Department of Clinical Neuroscience, Karolinska Institutet, SE-17177 Stockholm, Sweden; 5 Cancer Surveillance Branch, International Agency for Research on Cancer, F-69372 Lyon, France; brayf@iarc.who.int; 6 Department of Health Promotion, Sophiahemmet University, SE-114 86 Stockholm, Sweden; helio.manhica@ki.se; 7 Department of Global Public Health, Karolinska Institutet, SE-17177 Stockholm, Sweden; 8 Cancer Care Centre, Kilimanjaro Christian Medical Centre, Moshi P.O. Box 3010, Tanzania; serventifuraha@hotmail.com; 9 Department of Material Sciences, Norwegian University of Technology and Science, NO-7491 Trondheim, Norway; ragnhild.aune@ntnu.no; 10 Department of Epidemiology and Biostatistics, Institute of Public Health, Kilimanjaro Christian Medical University College, Moshi 2240, Tanzania; 11 Management and Development for Health (MDH), Dar es Salaam P.O Box 79810, Tanzania; 12 Division of Nursing, Department of Neurobiology, Care Sciences and Society, Karolinska Institutet, SE-17176 Stockholm, Sweden

**Keywords:** breast cancer, quality of life, reliability, validation, psychometric properties

## Abstract

Breast cancer is the most frequent cancer in women in Africa and contributes to premature death and poor quality of life. This study aimed to determine the validity, reliability, and psychometric properties of the Swahili version of EORTC QLQ-BR45 among women with breast cancer in Tanzania. A cross-sectional study design with non-probability convenience sampling was employed. Data were collected in two tertiary hospitals and one national cancer institute; 414 participants completed the EORTC-QLQ-C-30 and EORTC-QLQ-BR45. The reliability of QLQ-BR45 was measured using Cronbach’s alpha and McDonald’s Omega coefficients. The factor structure of EORTC QLQ-BR45 was assessed using confirmatory factor analysis. The internal consistencies for the five dimensions were all above 0.7 indicating satisfaction, except for systemic therapy side effects with a marginal value of 0.594 and significant correlations between the dimensions of QLQ-C30 and BR45. The final model fit well to the data, with the comparative fit index = 0.953, Tucker–Lewis index = 0.947, root mean square error of approximation = 0.041 (90% CI: 0.035, 0.046), and standardized root mean square residual = 0.072. In conclusion, the QLQ BR45 Swahili version displayed good reliability, validity, and psychometric properties and can be used in Swahili-speaking Sub-Saharan countries.

## 1. Introduction

Cancer in Sub-Saharan Africa (SSA) affects many of its one billion inhabitants. It is among the three leading causes of premature death (30–69 years) in almost all constituent countries [1]. Breast cancer is now the most frequent cancer in Africa and SSA, and 129,500 new cases were estimated in 2020 in the region and 64,000 deaths [2,3].

In Tanzania, a national cancer registration system coordinated by the Ministry of Health has been developed; existing data indicate increasing incidence rates, with 40,000 new cancer cases annually [4,5]. Female breast cancer is currently the second most common cancer in Tanzania, accounting for one in eight cancers diagnosed [6]. The incidence of breast cancer in Tanzania was 3037 and 1303 deaths in 2018, which is projected to increase by 120% in 2040 [7,8]. Of 2321 new cancer patients attending the Kilimanjaro Christian Medical Centre Cancer Care Centre [6] from December 2016 to October 2019, 15% were breast cancers [6]. 

Specialized cancer care in Tanzania is limited to five urban tertiary hospitals with limited services: two are located in Dar es Salaam, and one in Mwanza, Kilimanjaro, and Mbeya, respectively [9]. However, none of the primary healthcare levels where the majority resides offers cancer care services. Given the limited cancer services, there are unmet needs, and patients in Tanzania are likely to receive inadequate services, leading to a high mortality rate and poor quality of life [10]. Moreover, most patients are diagnosed at late stages III or IV [1,3,11]. Multiple factors could contribute to a late diagnosis, such as lack of screening, low education and health literacy, and low socio-economic status [12]. 

Quality of life (QoL) among breast cancer patients in Tanzania is low probably because of the limited cancer care centres. Previous investigators reported that cancer patients in Tanzania displayed low QoL in physical, social, and role functioning [13]. Moreover, the patients displayed a high level of emotional and cognitive function, but financial difficulties were most problematic for the patients, followed by pain. This critically highlights patients’ prevailing QoL and symptom severity in this group. The European Organization for Research and Treatment of Cancer (EORTC) developed and standardized EORTC-QLQ-C-30 to be used in all types of cancer studies for QoL, containing 30 items [14]. Over 5000 studies have used this instrument and it exists in over 100 languages www.qol.eortc.org (accessed on 2 July 2023). There are also different cancer-specific burden QoL questionnaires depending on the type of cancer to be studied. The first breast-cancer-specific questionnaire was EORTC-QLQ–BR23 developed in 1996 [15]. The evolution of diagnosis and treatment of breast cancer dramatically justified the need for an update to the EORTC-QLQ-BR23 [15]. In 2020, a new version with 22 additional items was developed, the EORTC QLQ-BR45 [16]. The EORTC QLQ-BR45 is standardized in 19 languages [17,18,19]. Few studies in Tanzania used EORTC-QLQ-C-30 and none utilized the EORTC-QLQ-BR23 or the BR-45 modules. 

The Kiswahili language, here called Swahili, is the national language in Tanzania and is one of the most widely spoken languages in Sub-Saharan Africa. It has more than 200 million speakers and is among the 10 most spoken languages in the world. Swahili is approved as an official language by the African Union [20], East African Community [21], and the Southern African Development Community [22] and is the first language in the African continent recognized by the United Nations Educational, Scientific and Cultural Organization [23]. Despite that, little has been done to utilize the existing resources to be translated into this language (Swahili) to communicate effectively within this region. Therefore, it is important to translate and validate questionnaires and communicate effectively with the target population, especially researchers who intend to gather and communicate information using this global official language. Breast cancer is increasing in Tanzania, in Sub-Saharan Africa, and in other regions where Swahili is spoken [3,24]. Most patients are, however, diagnosed at a late stage [25,26,27] and there is a lack of studies regarding QoL in women with breast cancer in Sub-Saharan Africa. Therefore, this study aimed to determine the QoL in women with breast cancer in Tanzania by translating, validating, determining the reliability, and establishing the psychometric properties of the Swahili version of the EORTC-QLQ-BR45.

## 2. Materials and Methods

### 2.1. Study Design and Setting

This was a descriptive cross-sectional design study where a non-convenience sampling was employed. Data were collected from November 2020 to August 2021 at three out of five cancer care centres in Tanzania, namely Kilimanjaro Christian Medical Centre Cancer Care Centre in Moshi (located in a zonal hospital), Ocean Road Cancer Institute in Dar es Salaam (located in a national hospital), and Bugando Medical Centre Cancer Care Centre in Mwanza (located in a zonal hospital). The COVID-19 pandemic had some negative impact on the data collection due to a decreased number of patients who were visiting healthcare facilities, although there was no restriction in Tanzania regarding travelling within the country. However, during the data collection, the research assistants and patients took the recommended COVID-19 precautions such as wearing masks and keeping distances according to the Tanzania government and WHO protocols.

### 2.2. Study Population

The study population consisted of adult women with breast cancer treated at one of the three cancer care centres in Tanzania, namely Kilimanjaro Christian Medical Centre Cancer Care Centre in Moshi, Ocean Road Cancer Institute in Dar es Salaam, and Bugando Medical Centre Cancer Care Centre in Mwanza. 

#### 2.2.1. Inclusion Criteria

Women aged 18–70 years, diagnosed with breast cancer, who could speak the Swahili language were asked to participate in the present study. 

#### 2.2.2. Exclusion Criteria

Terminal patients and those who could not understand or speak Swahili were excluded. 

#### 2.2.3. Recruitment Procedure

Convenience sampling was used to obtain the required sample across the three study settings after obtaining the ethical clearance certificate and permission from the respective hospitals’ executive directors. The researchers, thereafter, introduced the study to the hospitals’ matron who authorized access to patients at the wards and outpatient clinics. Women with breast cancer who visited the study setting for treatment were approached and informed about the study by the researcher and/or trained research assistants (who were also health care providers in the respective setting). Those who agreed to participate in the study were asked to sign an informed consent form to confirm their voluntary participation in the study. The research assistants assisted the participants by reading the questions and filling in the participants’ answers. This procedure was maintained throughout the course of the study. The reason why the research assistants read and filled in the questionnaires together with the participants was that there are still people in the country who are illiterate; therefore, to ensure the quality of the data, the researcher filled in the forms together with the respondents.

#### 2.2.4. Sample Size

The sample size was estimated using the sample size for a proportion formula, i.e., a planned value of p* = 0.5 [28]. Since most African countries have no national cancer registry, no clear proportion was given. A total of 422 patients was estimated. Convenience sampling was used to obtain the required sample; 414 (98% response rate) participants completed the questionnaires. This sample size complies with validation studies standards ranging from 100 to 400 [29]. 

### 2.3. Data Collection and Instruments

In this study, the Swahili versions of the EORTC-QLQ-C30 and the EORTC QLQ-BR45 were used. Permission to translate and use the tool was granted by EORTC headquarters in 2020. The EORTC QLQ-BR45 comprises 45 questions distributed into four functional scales (body image, sexual functioning, sexual enjoyment, and future perspective) and five symptom scales/items (systemic therapy side effects, breast symptoms, arm symptoms, and upset by hair loss). These tools use a four-point scale from 1 = not at all, to 4 = very much, and a scoring scale of 0–100, with a high score indicating better functioning and severity for high symptoms/item scale [13,29]. Data were collected by the researcher/research assistants, who administered the questionnaire to the patient after she had provided informed consent to participate in the study. The participant and the researcher or the research assistant sat in a quiet room together in the respective cancer care centre, where the survey was conducted. The researcher and/or research assistant read the questions and filled in the participant’s answers. The duration of the survey was 45 min to 1 h depending on the participant’s ability to understand and respond to questions.

### 2.4. Phase I: Cross-Cultural Adaption and Face Validity

The EORTC QLQ-BR45 was translated according to the EORTC translation unit standards [30]. The forward translations (English to Swahili) were completed by two translators who were native speakers of the Swahili language and fluent in the English language. Then, a reconciled translation was made based on the two translations; a third person with the same qualifications reviewed the translations and combined them into one reconciled version of the EORTC QLQ-BR45 in Swahili. The reconciled EORTC QLQ–BR45 Swahili version was translated into English by two independent persons who were fluent in English. The results of all steps (i.e., two forward translations, reconciliation, and two backward translations with comments) were put into a report, which allowed the EORTC translation unit to review the process. After the EORTC translation unit reviewed the report and confirmed the process, the translation was proofread by an external proofreader who audited the final version. Finally, the translated version was ready for linguistic validation (EORTC QLQ-BR45 Swahili version), the so-called pilot testing.

#### Piloting

The EORTC QLQ-BR45 Swahili version was piloted on a group of 10 breast cancer patients at Kilimanjaro Christian Medical Centre Cancer Care Centre who were native speakers of the Swahili language. According to the EORTC translation unit standards [30], the pilot was completed for 10 participants to pilot a face validation of the instrument. Small corrections to the questionnaire were made according to comments received in the pilot testing and forwarded to the EORTC. The translation was finalized and the EORTC QLQ-BR45 Swahili version was approved by the EORTC translation unit to be utilized in this project. No risk was observed throughout piloting for the participating patients, and they were not included in the study.

### 2.5. Phase II: Psychometric Evaluation

#### Statistical Analysis

For the current study, two approaches of psychometric analyses were considered: item response theory (IRT) and classic test theory (CTT) [31]. The CTT is the traditional approach to psychometric analysis that assumes the error score is random and independent of the true score. According to the CTT, the reliability of a test can be estimated by measuring the consistency of scores across test items. However, the CTT might be limited due to the high dependency on the sample characteristics [31], and IRT was developed to address this limitation and reduce its dependence on sample characteristics [32]. Several techniques belonging to CTT were, therefore, used to measure the psychometric properties of the EORTC QLQ-BR45 in the current study: internal consistency analysis, corrected item-total correlation, Confirmatory Factor Analysis (CFA), and assessment of measurement invariance. The reliability of the EORTC QLQ-BR45 was measured using Cronbach’s alpha and McDonald’s Omega coefficients [33] with values of 0.7 or higher indicating satisfaction [34]. A corrected item-total correlation was calculated to investigate the internal consistency of the EORTC QLQ-BR45 further. A corrected item-total correlation of >0.40 indicates that the item measures the same construct/domain as other items [34]. The factorial structure of the EORTC QLQ-BR45 was assessed using confirmatory factor analysis (CFA) with a diagonally weighted least squares (DWLS) estimator. Several structures/models were specified for the EORTC-QLQ-BR45. In the first model (Model A), the original structure of the EORTC QLQ-BR45 (i.e., three functional scales and 6 symptom scales) was assessed. In the next model (Model B), a two-dimensional structure of the EORTC QLQ-BR45 (i.e., one functional scale and one symptom scale) was tested. In Model C, the three-dimensional structure of the EORTC QLQ-BR45 (i.e., symptom scales as one-dimensional and therapy side effects as the latest dimension) was tested. Model D was tested based on the proposed prior five-factor structure from Tsui et al. [35], i.e., two functional scales and three symptom scales. In the final model, Model E, the final five-factor structure from Tsui et al. [35] was tested. The model fit was measured using the following indices: comparative fit index (CFI) and Tucker–Lewis index (TLI) >0.95, root mean square error of approximation (RMSEA) <0.08, and standardized root mean square residual (SRMR) <0.10 [36].

To further evaluate the EORTC QLQ-BR45 based on IRT, a Rasch analysis using a partial credit model was performed. Item scores were assessed using inlier-sensitive mean square (infit MnSq) and outlier-sensitive (outfit) MnSq with values within 0.5 and 1.5 as satisfactory [37]. Differential item functioning (DIF) was used to assess measurement invariance of the EORTC QLQ-BR45 across subgroups of the sample, including age (<50 vs. ≥50 years), menopausal status (yes vs. no), WHO performance status (scale 0 vs. scales 1–3), and cancer stage (stages 1–2 vs. stages 3–4). A DIF lower than 1 was considered a non-substantial DIF [38]. The EORTC-QLQ-C30 and QLQ-BR45 functional/item and symptoms mean scores were computed according to EORTC’s scoring manual [14,39]. The components of items were transformed into a range of 0–100 of which Cronbach’s alpha was computed.

The convergent validity of the EORTC QLQ-BR45 was examined by computing the Spearman’s rank correlation coefficients between the subscales of the EORTC QLQ-BR45 and EORTC-QLQ-C30. Known group validity was conducted to examine whether the EORTC QLQ-BR45 could distinguish between age (<50 vs. ≥50 years), WHO performance status scale [40] (scale 0 vs. scales 1–3), and cancer stage groups (stages 1–2 vs. stages 3–4). The Mann–Whitney U-test was used to compare the EORTC QLQ-BR45 subscale scores across age, WHO performance status, and cancer stage subgroups.

### 2.6. Ethical Considerations

An ethical clearance certificate was obtained from the National Institute for Medical Research number 3904 and from the Kilimanjaro Christian Medical University College Research Ethics and Review Committee number 2488. Permission was obtained from the executive directors of the respective study sites. Written informed consent was obtained from individual participants and participation was voluntary; the participants could withdraw their participation at any time without compromising their services. All data derived from the study is handled with confidentiality. The study follows the guidelines on research from the Declaration of Helsinki regarding research on human subjects [41].

## 3. Results

### 3.1. Sociodemographic and Clinical Characteristics of the Study Participants

A total of 414 participants were recruited with a mean age of 50.6 ± 10.7 years. The characteristics of the participants are shown in Table 1. Most participants had breast cancer at stages 3–4 (76.1%) and it was locally advanced (40.6%). More than half of the participants resided in urban areas (58.2%) and almost a third (30.4%) scored 0 on the WHO performance scale.

### 3.2. Psychometric Evaluation

The global health status/QoL of the participants was low with a mean score of 63.4 (SD = 20). Most of the functioning and symptoms subscales scores of Cronbach’s alpha for QLQ-C-30 were above the cut-off point > 0.70 except for systemic therapy side effects (α = 0.59) as displayed in Table 2.

The results of the CFA models for the EORTC QLQ-BR45 are displayed in Table 3. The original structure of the EORTC QLQ-BR45 (Model A) was not acceptable as evidenced by the poor model fit indices: CFI = 0.723, TLI = 0.696, RMSEA = 0.076 (90% CI: 0.073, 0.079), and SRMR = 0.08. Model B (two-dimensional structure of the EORTC QLQ-BR45 functional scales as one dimension and symptom scales as one dimension) yielded improved model fit indices but was still not acceptable: CFI = 0.816, TLI = 0.808, RMSEA = 0.129 (90% CI: 0.126, 0.131), and SRMR = 0.148. Similarly, the three-dimensional structure of the EORTC QLQ-BR45 (Model C) did not fit with the data: CFI = 0.819, TLI = 0.810, RMSEA = 0.128 (90% CI: 0.125, 0.131), and SRMR = 0.148. Model D was then tested based on Tsui et al.’s [35] recommended structure (i.e., a prior model of the five-dimensional structure of the EORTC QLQ-BR45). The model fit indices were acceptable: CFI = 0.956, TLI = 0.951, RMSEA = 0.075 (90% CI: 0.071, 0.079), and SRMR = 0. 097. In model E, the final five-dimensional structure recommended model by Tsui et al. [35] was examined. The final model (Model E) fits well with the data: CFI = 0.953, TLI = 0.947, RMSEA = 0.041 (90% CI: 0.035, 0.046), and SRMR = 0.072. All factor loadings were significant and ranged from 0.10 to 0.78. Model E was chosen to proceed further because it had higher fit indices compared to model D.

Additionally, the factor covariances among the EORTC QLQ-BR45 were all significant and ranged from −0.177 (between systemic therapy side effects and sexual functioning and enjoyment) to 0.796 (between systemic therapy side effects and endocrine therapy symptoms). The Cronbach’s alpha internal consistencies for the five dimensions of the EORTC QLQ-BR45 were all above 0.7, except for systemic therapy side effects, which had a marginal value of 0.594 (Table 4). The results of the Rasch analysis are presented in Table 4. The item difficulty ranged from −1.41 (SX45: Sexually active) to 1.08 (ET69: Has weight gain been a problem for you?). The infit MnSQ and outfit MnSq of all items were within the acceptable range of 0.5 to 1.5. No significant differential item functioning (DIF) was found across age, menopausal status, performance status, and cancer stage subgroups (DIF ≤ 1.0). However, the patients with the low cancer stage reported significantly low difficulty (DIF = −1.34) in understanding item 45 (Have you been sexually active (with or without intercourse))?

There were significant correlations between the dimensions/domains of the EORTC QLQ-BR45 and EORTC QLQ-C30 (Table 5). However, sexual functioning and enjoyment were not significantly correlated with emotional, cognitive, and social functioning as well as fatigue, nausea, pain, and global health status. The magnitude of the significant correlation was not that high as only a few were strong at greater than ±0.5.

The results of the known group validity of the EORTC QLQ-BR45 are shown in Table 6. Body image and sexual functioning significantly differentiated between patients with young and old ages (i.e., ≥50, <50). On the other hand, patients with high and low performance reported significantly different scores in the Systemic therapy side effects, arm and breast symptoms, and endocrine therapy symptoms domains. No significant differentiation was found in EORTC QLQ-BR45 domains except for a marginally significant *p*-value for body image (*p* = 0.048).

## 4. Discussion

The study was conducted to meet the international standards of measuring the quality of life among breast cancer patients in Tanzania using the standardized EORTC QLQ-BR45 Swahili version by assessing its validity and reliability. This study shows that the Swahili version of EORTC QLQ-BR45 has good reliability, validity, and psychometric properties. To ensure the quality of the EORTC QLQ-BR45 Swahili version is culturally sensitive, the English version was translated into Swahili according to EORTC translation guidelines [30]. First, the English version of EORTC QLQ-BR45 was translated forward and backwards into Swahili to attain the final EORTC QLQ-BR45 Swahili version, which was piloted. Difficult words that emerged during piloting were amended, it was worded [19] to ensure that it was linguistically and culturally acceptable to all levels of education in the field, and finally, the tool was approved by the EORTC translation unit.

The EORTC QLQ-BR45 English and Swahili version instruments validation approach utilized a similar QoL questionnaire with 45 items. The EORTC QLQ-BR45 Swahili version was used for the first time, it was validated, and psychometric properties were computed. Our findings are in congruence with the original version of EORTC QLQ-BR45 English version, as factorial loading was significant [16]. Moreover, most dimensions had an acceptable internal consistency similar to other contexts [18,19] except for Systemic therapy side effects. This implies the format, structure, wording, comprehension, and items of the questionnaire fulfilled the required standard of translation to meet the cultural context of the Swahili language. However, this demonstrated robust validity and reliability of the items forming most of the dimensions trusted to measure breast cancer patients’ quality of life in Swahili-speaking communities. Moreover, systemic side effects imply nutrition uptake and physical body deterioration, thus patients are likely to score low.

Moreover, there were significant correlations between dimensions of the Swahili version of EORTC QLQ-BR45 and EORTC QLQ-C30, though sexual functioning and enjoyment were not statistically significantly correlated with emotional, cognitive, or social functioning as well as fatigue, nausea, pain, or global health status. The findings could probably be attributed to African countries’ culture, including Tanzania, whereby people are shy or prohibited from discussing sexual matters openly, and thus a minimal response to the questions related to sexuality occurred, which affects the statistical test. This concurs with [18], though in Ehab et al. [17], most patients refused to respond to questions related to sexual matters. This cultural context affects the community, especially for the chronically or terminally ill including cancer patients who, in most cases, sexual desire declines as induced by the cancer disease and treatment [42], resulting in psychological torture due to low health literacy of underlying body pathological changes. Moreover, health workers lack the skills to recognize sexual disorders in their clients or patients [43], accelerating minimal assistance, and this could induce psychological effects in this patient group [44].

The results of known group validity of the EORTC QLQ-BR45 demonstrated body image and sexual functioning significantly differentiated among age groups with a high burden in old age. Young women aged <50 years had better functioning compared to their counterparts. This demonstrated that the Swahili version is valid and reliable as most theories discriminate the performance and severity of diseases among age groups, with poor performance in advanced diseases and a high burden among older ages.

The CFA was computed to assess the EORTC QLQ-BR45 Swahili version’s structure. Several models were examined to test scale structure via the CFA. The final model demonstrated acceptable fit indices of the EORTC QLQ-BR45 Swahili version structure with CFI and TLI >0.90, similar to other studies [18,19,35]. The final model fit fulfilled the parameters and values and is, therefore, suitable to be used in Swahili-speaking geographical areas to measure QoL among breast cancer patients.

The global quality of life score of the EORCT QLQ-BR45 and QLQ C-30 Swahili version among breast cancer patients in Tanzania was low, being 63 (SD = 20). This could probably be due to a late diagnosis [45] and low socio-economic status [12]. Moreover, continuity of care among cancer patients in Tanzania after discharge from the cancer care centre is a big challenge, as most of the patients rely upon primary healthcare facilities with non-oncology specialities [10,11,25]. This implies inadequate services with physical and psychological effects on patients, thus leading to low quality of life in this patient group. Furthermore, the inaccessibility of cancer services in Tanzania is a major challenge for cancer patients [10,11,17,46]. However, the government made efforts to access healthcare facilities within 5 km [5], though they are still struggling to invest in cancer services at primary healthcare facilities and regional referral hospitals, so there is a long journey to improve cancer services and QoL for this patient group.

### Strengths and Limitations

This study utilized data from cancer centres in two (2) zonal referral hospitals and one (1) national referral hospital in Tanzania. Therefore, the sample can be considered representative of the entire country. This ensures the reliability of the data collected in this research project. Moreover, the sample size was large enough to compute the psychometric properties of the EORTC QLQ-BR45 Swahili version. Confirmatory factor analysis demonstrated strong scale structure and overall internal consistency for functioning and the symptoms scale meets the international standards set for measuring QoL among breast cancer patients. In this context, the researcher recommends the EORTC QLQ-BR45 Swahili version be used to measure QoL among breast cancer patients globally as an evaluation of treatment outcomes and strategies to improve survival rates. The inadequate response rate for sexual functioning and sexual enjoyment limited the evaluation of the correlation between dimensions and some EORTC QLQ C-30 functioning and symptoms domains, demanding advocacy for sexual education both to patients and healthcare providers to assist patients, as both breast cancer disease and treatment affect sexual desire. Sexual dysfunction among these groups can affect the quality of life in addition to the cancer disease and treatment, thus this needs to be taken care of.

## 5. Conclusions

The Swahili version of EORTC QLQ-BR45 has good psychometric properties, is reliable and valid, and can be used to determine breast cancer patients’ QoL in Swahili-speaking Sub-Saharan African countries. In this study, we believe using the EORTC QLQ-BR45 Swahili version will simplify data collection in this region and measure QoL among breast cancer patients accurately will assist in improving the QoL of patients with breast cancer. Further studies that measure the QoL during breast cancer treatment are recommended to confirm the results of the present study.

## Figures and Tables

**Table 1:** Baseline characteristics of participants *n* = 414.

Sociodemographic and Clinical Characteristics		Clinical Characteristics	
Variable	Frequency (%)	Variable	Frequency (%)
***Site***		***Cancer stage***	
KCMC	140 (33,8)	Stage 1	12 (2.1)
ORCI	140 (33.8)	Stage 2	80 (19.3)
BMC	134 (32.4)	Stage 3	127(30.7)
	***Mean (SD)***	Stage 4	188 (45.4)
***Age (Years)***	50.57 (10.7)	In-situ	1 (0.2)
<40	76(18.4)		***Mean (SD)***
40–49	112(27.1)	***Tumor size in cm***	6.3 (7.0)
50–59	105(25.4)	***Duration of disease since diagnosis (Months)***	17.28 (21.98)
60–69	108(26.1)	*Co-morbidities*	
>70	13(3.1)	Hypertension	97 (23.4)
***Marital status***		Diabetes mellitus	13 (3.1)
Single	59 (14.3)	HIV	16 (3.9)
Cohabiting	3(0.7)	TB	2 (0.5)
Married	251 (60.6)	Hypertension + Diabetes mellitus	12(2.9)
Divorced	20 (4.8)	Others	8 (1.9)
Widow/widower	72 (17.8)	None	265(64.0)
***Participants Have Children***		***Disease pattern***	
Yes	379 (91.5)	Local	65 (15.7)
No	31(7.5)	Locally advanced	168 (40.6)
***Educational Level***		Metastatic	170 (41.1)
Never attended	32 (7.7)	***Hormonal factor and HER2-receptor status***	
Primary	224 (51.4)	ER	36 (8.7)
Secondary	92(22.2)	PR	8 (1.9)
Vocational School	45 (10.9)	HER2	152 (36.7)
University	16 (3.9)	ER + PR	43 (10.4)
***Occupation***		ER + HER2	11 (2.7)
Public/Governmental employment	44(10.6)	PR + HER2	9 (2.2)
Private sector employment	36(8.7)	ER + PR + HER2	23 (5.6)
Self-employment	127(30.7)	Triple-negative	34 (8.2)
Unemployed	199(48.1)	Not reported	26 (6.3)
***Residence***		***Tumor grade***	
Urban	241(58.2)	Grade I: Well differentiated	28 (6.8)
Rural	171(43.2)	Grade II: Moderately differentiated	114 (27.5)
***Menopause status***		Grade III: Poorly differentiated	57 (13.8)
Yes	226 (54.6)	Not reported	108 (26.1)
No	187 (45.2)	***WHO Health Status Performance Scale***	
***Patient Diagnosis Status***		Scale 0	126 (30.4)
New	54 (14.3)	Scale 1	84 (20.3)
Follow up	223 (53.9)	Scale 2	27 (6.5)
Recurrence	120 (29.0)	Scale 3	6 (1.4)

**Table 2:** QLQ-C30 Subscale and internal consistency score *n* = 414.

	Subscale Scores (0 to 100) *	
		Internal Consistency
Instrument Subscale	Mean ± SD	Cronbach’s Alpha
QLQ C30		
***Global health status/QoL***	63.4 ± 20.0	0.84
Physical functioning (PF)	71.6 ± 25.4	0.85
Role functioning (RF)	66.9 ± 33.5	0.91
Emotional functioning (EF)	75.9 ± 24.7	0.84
Cognitive functioning (CF)	81.9 ± 22.5	0.57
Social functioning (SF)	66.3 ± 32.1	0.80
Fatigue (FA)	27.9 ± 23.6	0.73
Nausea, vomiting (NV)	16.1 ± 22.4	0.72
Pain (PA)	32.7 ± 28.6	0.74
***Single item sub-scales***		
Dyspnea (DY)	12.3 ± 25.2	n.a
Insomnia (SL)	26.3 ± 31.8	n.a
Appetite loss (AP)	30.1 ± 33.9	n.a
Constipation (CO)	12.5 ± 24.5	n.a
Diarrhea (DI)	06.6 ± 39.4	n.a
Financial problems	60.1 ± 39.4	n.a

* Higher Scores for Symptoms Imply More Severe Symptoms, While Higher Scores for Functioning Imply Greater Ability. n.a: Not applicable.

**Table 3:** Confirmatory factor analysis models.

	*χ*^2^ (df)	CFI	TLI	SRMR	RMSEA (90% CI)
**Model A**	2661.022 (783)	0.723	0.696	0.0755	0.076 (0.073–0.079)
**Model B**	7403 (944)	0.816	0.808	0.148	0.129 (0.126–0.131)
**Model C**	7297 (942)	0.819	0.810	0.148	0.128 (0.125–0.131)
**Model D**	1404.822 (424)	0.956	0.951	0.097	0.075 (0.071–0.079)
**Model E**	618.784 (367)	0.953	0.947	0.072	0.041 (0.035–0.046)

Model A = All items in original dimension. Model B = two-dimensional structure of the BR45 functional scales as one dimension, symptoms scales as one dimensional. Model C = three-dimensional structure of the BR45 functional scales as one dimension, symptom scales as one dimensional and therapy side effect as the latest dimension. Model D = Tsui’s et al. [35] prior 5-dimensional structure model. Model E = final Tsui’s et al. [35] recommended model.

**Table 4:** Psychometric properties of the EORTC QLQ-BR45 at the item level.

Item #	Analyses from Classical Test Theory				Rasch Analyses						
	Factor Loading *^,†^	Corrected Item Total Correlation	Cronbach’s Alpha	McDonald’s Omega Coefficient	Infit MnSq	Outfit MnSq	Difficulty	DIF Contrast across Age ^§,¶^	DIF Contrast across Menopausal Status ^§,#^	DIF Contrast across Performance Status ^§,#^	DIF Contrast across Cancer Stage ^§,#^
*Systemic therapy side effects*			0.594	0.571							
SYS31	0.358	0.273			1.17	1.20	0.63	−0.37	0.29	−0.13	0
SYS32	0.335	0.479			0.78	0.75	−0.36	0	0.15	−0.15	0.04
SYS33	0.417	0.377			1.05	1.06	0.97	−0.14	0.23	−0.10	−0.09
SYS34	0.096	0.262			1.24	1.30	−1.21	0.27	−0.32	−0.06	−0.03
SYS36	0.537	0.328			0.84	1.04	0.10	−0.11	0.09	0.03	0.11
SYS38	0.374	0.333			0.83	1.03	−0.13	0.10	−0.16	0.25	−0.08
*Body image*			0.876	0.876							
BI39	0.794	0.734			0.98	1.03	−0.59	0.44	−0.11	0.06	−0.27
BI40	0.693	0.785			0.84	0.77	0.52	−0.08	−0.38	−0.34	0
BI41	0.677	0.683			1.20	1.19	−0.44	0	0.26	0.12	0.20
BI42	0.633	0.737			0.97	0.95	0.52	−0.44	0.20	0.10	0.10
*Sexual functioning and enjoyment*			0.851	0.877							
SX44	0.785	0.795			0.82	0.82	1.04	0.03	−0.31	0.75	0.02
SX45	0.403	0.640			1.25	1.37	−1.41	0.23	0.49	−1.34	0.25
SE46	0.705	0.777			0.92	0.92	0.37	−0.24	−0.09	0.30	−0.27
*Arm and breast symptoms*			0.824	0.824							
ARM47	0.661	0.568			0.98	0.96	−0.56	−0.39	0.02	0.05	0.10
ARM48	0.453	0.494			1.38	1.13	0.87	−0.48	0.24	0.08	−0.25
ARM49	0.476	0.516			1.21	1.10	0.40	−0.06	0.16	−0.06	0.16
BR50	0.686	0.681			0.68	0.69	−0.62	0.19	0.13	0.06	0.20
BR51	0.564	0.633			0.92	0.84	−0.32	0.14	−0.06	−0.39	−0.23
BR52	0.500	0.617			0.92	0.89	0.48	0.19	−0.21	0.63	−0.14
BR53	0.470	0.470			1.16	1.17	−0.26	0.25	−0.21	−0.15	0
*Endocrine therapy symptoms*			0.780	0.763							
SYS37	0.500	0.415			1.16	1.02	−0.44	−0.15	0	−0.57	−0.48
ET54	0.429	0.428			1.20	1.14	−0.23	0	0	−0.48	−0.44
ET63	0.443	0.563			0.80	0.79	−0.11	−0.23	−0.17	0.24	−0.03
ET64	0.410	0.531			0.89	0.87	0.25	−0.13	0.18	0.01	0.20
ET65	0.473	0.586			0.71	0.75	−0.34	0.07	−0.06	0.09	−0.04
ET66	0.388	0.577			0.97	0.70	0.20	0	0.18	0.11	0.24
ET67	0.434	0.501			0.88	0.95	−0.34	−0.05	0.20	0.03	0.18
ET68	0.163	0.228			1.41	1.45	−0.07	0.48	−0.25	0.71	0.31
ET69	0.211	0.397			1.30	0.91	1.08	0	−0.08	0.10	0.37

* All factor loadings were significant at 0.001. ^†^ Based on the first-order confirmatory factor analysis (CFA). ^§^ DIF contrast > 1 indicates substantial DIF. ^¶^ DIF contrast across age = Difficulty for younger patients (<50)-Difficulty for older patients (≥50). ^#^ DIF contrast across menopausal status = Difficulty for non-menopause patients -Difficulty for menopause patients. ^#^ DIF contrast across performance status = Difficulty for patients with low-performance status (scale 0) − Difficulty for patients with high-performance status (scale 1–3). ^#^ DIF contrast across cancer stage = Difficulty for patients with low cancer stage (1-2) − Difficulty for patients with high cancer stage (3–4). MnSq = mean square error; DIF = differential item functioning.

**Table 5:** Correlations between the domains of the QLQ-C30 and QLQ-BR45.

QLQ-C30 Domain	Physical Functioning	Role Functioning	Emotional Functioning	Cognitive Functioning	Social Functioning	Fatigue	Nausea and Vomiting	Pain	Global Health
**QLQ-BR45** Systemic therapy side effects	−0.283 **	−0.271 **	−0.288 **	−0.312 **	−0.330 **	0.503 **	0.425 **	0.356 **	−0.325 **
Body image	0.214 **	0.185 **	0.409 **	0.254 **	0.241 **	−0.278 **	0.037	−0.260 **	0.157 **
Sexual functioning and enjoyment	−0.118 *	−0.139 **	−0.011	−0.083	−0.033	0.033	−0.015	−0.033	−0.056
Arm and breast symptoms	−0.498 **	−0.516 **	−0.445 **	−0.418 **	−0.516 **	0.565 **	0.232 **	0.580 **	−0.345 **
Endocrine therapy symptoms	−0.394 **	−0.371 **	−0.405 **	−0.444 **	−0.415 **	0.495 **	0.217 **	0.476 **	−0.199 **

* Significance level < 0.05. ** Significance level < 0.01.

**Table 6:** Known group difference of the QLQ-BR45 subscale scores by age, performance status, and cancer stage.

	Age			WHO Performance Status			Cancer Stage		
Domain	≥50 Mean ± SD	<50 Mean ± SD	*p*-Value	Scale 0 Mean ± SD	Scales 1–3 Mean ± SD	*p*-Value	Stages 1–2 Mean ± SD	Stages 3–4 Mean ± SD	*p*-Value
Systemic therapy side effects	26.19 ± 18.06	26.33 ± 17.48	0.946	25.84 ± 13.49	33.48 ± 15.96	<0.001	23.79 ± 18.20	26.87 ± 17.73	0.159
Body image	81.26 ± 25.25	76.05 ± 24.48	0.004	84.66 ± 16.81	81.05 ± 23.86	0.774	74.64 ± 26.61	79.97 ± 24.50	0.048
Sexual func-tioning and enjoyment	8.65 ± 15.96	25.38 ± 25.15	<0.001	22.57 ± 23.26	20.13 ± 23.43	0.394	81.84 ± 24.75	16.19 ± 21.85	0.644
Arm and breast symptoms	21.75 ± 20.14	20.50 ± 20.16	0.480	13.49 ± 13.86	24.09 ± 20.01	<0.001	16.98 ± 15.87	22.46 ± 21.22	0.088
Endocrine therapy symptoms	16.30 ± 15.77	14.70 ± 14.20	0.370	11.40 ± 11.42	16.09 ± 13.50	<0.001	15.38 ± 14.48	15.48 ± 15.31	0.666

## Data Availability

The data presented in this study are available on request from the corresponding author.
